# The Promise and Challenges of Intensive Longitudinal Designs for Imbalance Models of Adolescent Substance Use

**DOI:** 10.3389/fpsyg.2018.01576

**Published:** 2018-08-28

**Authors:** David M. Lydon-Staley, Danielle S. Bassett

**Affiliations:** ^1^Department of Bioengineering, University of Pennsylvania, Philadelphia, PA, United States; ^2^Department of Electrical and Systems Engineering, University of Pennsylvania, Philadelphia, PA, United States; ^3^Department of Neurology, Perelman School of Medicine, University of Pennsylvania, Philadelphia, PA, United States; ^4^Department of Physics & Astronomy, College of Arts and Sciences, University of Pennsylvania, Philadelphia, PA, United States

**Keywords:** imbalance model, risk-taking, substance use, intensive longitudinal designs, adolescence

## Abstract

Imbalance models of adolescent brain development attribute the increasing engagement in substance use during adolescence to within-person changes in the functional balance between the neural systems underlying socio-emotional, incentive processing, and cognitive control. However, the experimental designs and analytic techniques used to date do not lend themselves to explicit tests of how *within-person change* and *within-person variability* in socio-emotional processing and cognitive control place individual adolescents at risk for substance use. For a more complete articulation and a more stringent test of these models, we highlight the promise and challenges of using intensive longitudinal designs and analysis techniques that encompass many (often >10) within-person measurement occasions. Use of intensive longitudinal designs will lend researchers the tools required to make within-person inferences in individual adolescents that will ultimately align imbalance models of adolescent substance use with the methodological frameworks used to test them.

Substance use during adolescence sets the stage for future substance use and abuse (U.S. Department of Health and Human Services, 2012). From the perspective of imbalance models of risk-taking (Casey and Jones, 2010; Lydon et al., 2015), adolescents are particularly vulnerable to drug use due to normative increases in the activity of limbic and paralimbic regions involved in socio-emotional processes such as incentive processing (Ernst et al., 2005; Galvan et al., 2006) alongside continued immaturities in the functioning of prefrontal regions involved in cognitive control (Hwang et al., 2010; Ordaz et al., 2013). This configuration results in an *imbalance* of limbic relative to prefrontal control that renders adolescents more sensitive to rewarding stimuli and less likely to inhibit impulses to approach rewards relative to children and adults (Somerville et al., 2011). Adolescents with the greatest imbalance between limbic relative to prefrontal control are thought to be particularly vulnerable to substance use during this period of heightened risk (Bjork and Pardini, 2015). Models have become more nuanced over time, broadening the scope to consider pathways to drug use beyond self-control failures in the face of potential rewards (Geier, 2013; Romer et al., 2017). However, the core of these models maintains a key role for functions associated with the socio-emotional and cognitive control systems, as well as system-by-system interactions, in explaining adolescent risk-taking and substance use (Casey et al., 2016).

Empirical studies of imbalance models of adolescent substance use have been the subject of numerous reviews (Richards et al., 2013; Smith et al., 2013; Lydon et al., 2014; Bjork and Pardini, 2015), which collectively indicate that drug use is associated with between-person differences in traits, behaviors, and neurobiological features associated with the functioning of the socio-emotional and cognitive control systems. Between-person differences in changes in these features are also associated with changes in drug use through adolescence. The designs and analytic techniques used to date, however, are limited in the extent to which they can appropriately test the *within-person* mechanisms proposed by imbalance models. Here we offer an overview of this mismatch between propositions of imbalance models and the methods used to test them, followed by a discussion of potential future advancements through the use of intensive longitudinal designs.

## Intraindividual Change and Intraindividual Variability in Individual Adolescents

**FIGURE 1:**
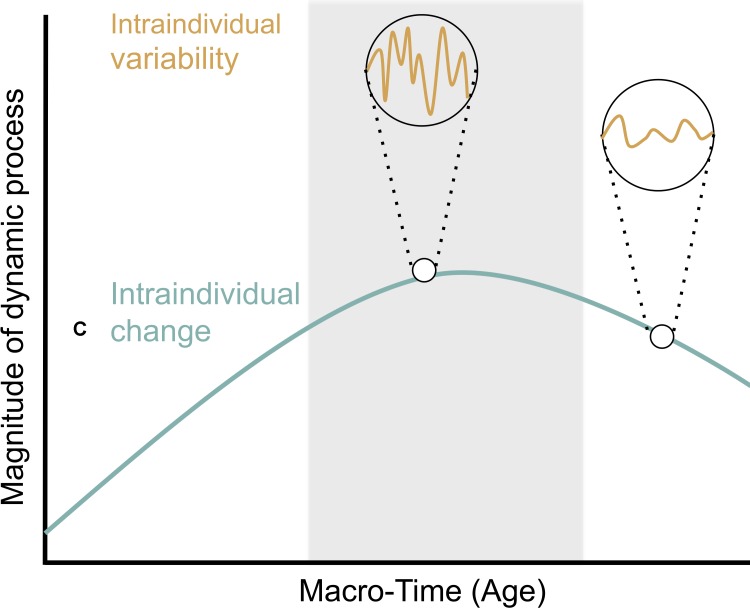
Intraindividual change and intraindividual variability (figure adapted from Ram and Gerstorf, 2009; with permission from the authors). Intraindividual change (green line) describes within-person change resulting from long-term processes over the course of years. Intraindividual variability (orange line) refers to changes occurring on relatively shorter timescales that are conceived of as fluctuations resulting from short-term processes over the course of seconds, minutes, days, and weeks. Differences between the two circles highlight that the extent of intraindividual variability may change with age. The adolescent period is highlighted by a gray background and is marked by a peak in the magnitude of the dynamic characteristic under consideration. This peak was chosen to reflect findings of increased risk-taking and sensation seeking during adolescence relative to childhood and adulthood (e.g., Burnett et al., 2010). Further, greater intraindividual variation is portrayed during adolescence relative to other periods. This reflects findings of greater variability in risk-taking in adolescents relative to children in recent work (e.g., McCormick and Telzer, 2017).

Imbalance models highlight the importance of *intraindividual change* and *intraindividual variability* in socio-emotional and cognitive control system functioning for understanding change in drug use. Intraindividual change refers to within-person change resulting from long-term processes, such as gradual brain maturation over the course of years. Intraindividual variability, in contrast, refers to transient, within-person fluctuations occurring on relatively shorter timescales (Nesselroade, 1991; Ram and Gerstorf, 2009; **Figure 1**).

Describing intraindividual change will facilitate the direct testing of imbalance models given their focus on brain structure and function as explicative of drug use. Notice, for example, the focus on within-person change in individual adolescents in this excerpt summarizing imbalance model research on adolescence in order to inform the workings of the juvenile justice system (Steinberg et al., 2008, p. 1765, italics added for emphasis):

“There is … compelling neurobiological evidence for *changes* in brain structure and function during adolescence and early adulthood that facilitate improvements in self-regulation that *permit individuals to modulate their inclinations* to seek rewards … ”.

It is the capacity to make inferences linking within-person change in brain structure and function to improvements in self-regulation in individuals that makes the most compelling argument for a focus on the neurobiological level of analysis here.

In addition, describing intraindividual variability in adolescent functioning will be required to fully articulate imbalance models of adolescent drug use. The earliest iterations of imbalance models emphasized that a decision-making style promoting risk-taking is not a stable characteristic of adolescents but is instead a variable characteristic, emerging in situations that arouse the socio-emotional system (Casey et al., 2008). In line with this perspective, adolescent drug use shows marked day-to-day variability (Arbel et al., 2016), laboratory tasks indicate that adolescent risk-taking is especially likely to be observed in the presence of peers and in emotionally-salient contexts (Figner et al., 2009; Chein et al., 2011), and adolescents are more likely than children to change their risk-taking behavior from trial to trial in response to feedback (McCormick and Telzer, 2017).

## Aligning Models and Methods Using Intensive Longitudinal Designs

The mismatch between the intraindividual change and intraindividual variability highlighted in imbalance models and the between-person inferences allowed by current methods has not gone unnoticed in previous work. Indeed, the limitations section of many manuscripts call for longitudinal designs and analysis techniques in order to allow for within-person inferences. Longitudinal designs, however, must also be coupled with appropriate analysis techniques (see King et al., 2017; Madhyastha et al., 2017) and, further, *intensive* longitudinal designs and analyses will be necessary to determine the role of imbalance model mechanisms in adolescent drug use.

Intensive longitudinal designs is an umbrella term that subsumes a variety of terms used to describe methods that employ repeated assessments of individuals, sometimes *in situ*, using an array of technologies, including daily diaries and smartphones (Bolger and Laurenceau, 2013). The density of time points required for data to be considered intensive longitudinal data varies widely: a minimum of five occasions (Bolger and Laurenceau, 2013), tens of measurement occasions (Walls and Schafer, 2006), or at least 20 occasions (Collins, 2006). Intensive longitudinal designs can span multiple time frames and are necessary for analyzing the unfolding of behavior over the course of seconds (e.g., changes in the configuration of functional brain systems during cognitive control performance; Braun et al., 2015), days (e.g., changes in adolescent mood and family functioning; Fosco and Lydon-Staley, 2017), weeks (e.g., training-related improvements in cognitive ability; Schmiedek et al., 2010), and years (e.g., the pubertal process; Marceau et al., 2011).

Different designs are variably amenable to capturing processes unfolding at different timescales. Laboratory assessments, for example, can be designed and analyzed to provide insight into processes unfolding over the course of milliseconds (Teslovich et al., 2014). Daily diary designs (Bolger et al., 2003), in which participants complete end-of-day reports on their behaviors and experiences that day for a period of days or weeks, capture day-to-day changes in processes. Experience-sampling designs (Hektner et al., 2007), in which participants complete brief surveys about their immediate states and environments several times a day, capture a finer timescale relative to daily diaries. Supplementing panel designs familiar to developmental psychologists consisting of relatively few measurement occasions (often approximately three or fewer separated by many months and often years) with more frequent assessments also results in data that may be considered intensive longitudinal data, as they allow for inferences of intraindividual change. Below we highlight how intensive longitudinal designs may be leveraged to overcome the mismatch between models and methods in the context of imbalance models of adolescent drug use. Throughout, we highlight challenges associated with the use of intensive longitudinal designs.

## Matching Models and Methods: Capturing Intraindividual Change

Few longitudinal investigations of intraindividual change in imbalance model components and how they relate to adolescent drug use exist. The majority of studies have been cross-sectional, limiting what we can say about the effect of development on drug use. Longitudinal data by itself will be insufficient to test the importance of intraindividual change in imbalance model features for understanding substance use. Paulsen et al. (2015), for example, examined developmental change in incentive-motivational circuitry longitudinally using an anti-saccade task. Youth aged 10–22 years were sampled two to three times over approximately 15 months intervals in an accelerated longitudinal design. Accelerated longitudinal designs greatly reduce the measurement collection time and result in the collection of data spanning age ranges required to test theories of adolescent development. However, the parameters of interest, in this case the linear and quadratic trends in incentive processing with age, represent population average rates of change (Galbraith et al., 2017). With two or three time points per person, it is not possible to describe intraindividual change through adolescence because there are insufficient data to accurately describe any single individual.

Collection of intensive longitudinal data results in sufficient data points per individual that researchers can model intraindividual change. The consideration of intraindividual change, as well as requiring enough data to capture within-person change, requires appropriate analytic techniques. Imagine, for example, that we have collected data on both sensation seeking and alcohol use across 10 waves at 3 months intervals, and we wish to know how within-person change in sensation seeking is associated with change in an individual’s alcohol use. We might model the data using a multivariate latent growth curve (Grimm et al., 2016) and examine the association between the sensation seeking slope and the alcohol-use slope. If the association between these two slopes is positive, then as sensation seeking increases, alcohol use also increases. However, only a between-person inference is possible here. The slope parameters, despite being made up of repeated measures within individuals, are between-person descriptions of rates of change. Thus, our conclusion is that people with greater increases in sensation seeking have greater increases in alcohol use. This is not the inference that we often set out to make.

**FIGURE 2:**
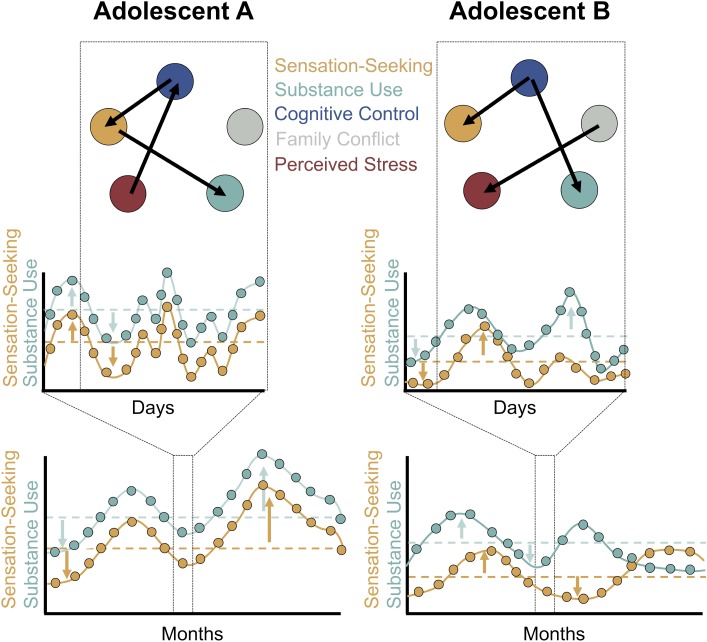
A sampling of the multiple timescales of sensation seeking and substance use. Data from two hypothetical adolescents are presented. Intensive longitudinal data on sensation seeking and alcohol use collected several weeks apart provide insight into intraindividual change in sensation seeking and substance use **(bottom)**. The data simultaneously contain information on between-person variation (the dashed horizontal lines indicating that Adolescent A exhibits higher levels of both sensation seeking and alcohol use than Adolescent B, on average) and within-person variation (the arrows indicating deviations from between-person means). Once within-person and between-person variation are disaggregated (see Curran and Bauer, 2011), multilevel models may be employed to make within-person inferences about how intraindividual change in sensation seeking (i.e., increases in sensation seeking relative to one’s own mean level of sensation seeking) is associated with change in one’s substance use behaviors. Daily diary and experience-sampling designs provide insight into intraindividual variability – or transient, within-person changes – in sensation seeking and substance use at the daily or finer timescale **(middle)**. These data may be treated in a similar fashion to the intensive longitudinal data in the bottom panel but the inferences now relate to transient fluctuations and processes at shorter timescales. One might ask if levels of substance use are greater than usual on days when an adolescent’s sensation seeking is higher than usual. With the dense data streams available through experience-sampling designs, moment-to-moment associations may be modeled as person-specific networks **(top)**. Here, the data from Adolescent A and Adolescent B are not pooled in a multilevel model. Instead, circles represent constructs of interest to imbalance models and broader biopsychosocial constructs. Arrows represent temporal associations among constructs. For example, the arrow from cognitive control to sensation seeking indicates that in moments when cognitive control is higher than usual for both Adolescent A and Adolescent B, sensation seeking is blunted. Notably, patterns specific to individuals may emerge: see for instance the differing pattern of associations among cognitive control and substance use across both networks.

To determine what happens to an individual’s level of drug use when that individual’s level of sensation seeking increases, we must disaggregate the between-person and within-person components of change over time, as repeated measures data simultaneously contain information on between-person and within-person variation (Curran and Bauer, 2011). There are a number of ways to perform this disaggregation. Typically, time-dependent variables are calculated as deviations from person-level means. Once this disaggregation is complete we can make inferences about intraindividual change, examining how an adolescent’s usual level of alcohol use might differ during periods when that adolescent’s sensation seeking tendencies are higher than usual (**Figure 2** bottom).

Capturing intraindividual change would bring us closer to examining how traits, behaviors, and neurobiological features associated with the functioning of the socio-emotional and cognitive control systems are associated with changes in substance use. Challenges associated with capturing intraindividual change include the greater monetary costs associated with the collection of data at many more timepoints and the increased respondent burden. Methods to identify measures that are maximally predictive of outcomes of interest are emerging (Brick et al., 2017). These methods will allow for the construction of shorter assessments to balance the more frequent assessments associated with intensive longitudinal designs.

## Matching Models and Methods: Capturing Intraindividual Variability

Imbalance models highlight intraindividual variability as a core feature of adolescent behavior. Yet, little work addresses the question of how within-person variability in imbalance model processes relates to drug use. Our understanding of the timescales at which the functions highlighted by imbalance models vary within person is limited by the scarcity of intensive longitudinal data with assessments of socio-emotion and cognitive control functioning in adolescents. Evidence exists for within-person variability in adolescent cognitive control (Rahdar and Galván, 2014), risky decisions (Goldenberg et al., 2016), and functional Magnetic Resonance Imaging (fMRI) indices of socio-emotional processes (Chein et al., 2011). These data motivate the collection of intensive longitudinal data, from which we may assess the degree to which an individual can be thought of as a dynamic, labile, and fluctuating entity, thereby adequately capturing a component of individual functioning long-held to be essential for a thorough characterization of the developing individual (Nesselroade, 1991). As well as providing a more complete picture of the developing adolescent, emerging evidence suggests that transient, within-person changes in functions highlighted by imbalance models may be important for understanding substance use. Goldenberg et al. (2016), for example, observed that adolescents exhibiting greater response variability on a risk-taking task also had lower perceptions of real-world risks, including drug use.

Although research in the field has captured fluctuations in cognitive control and socio-emotional processing on the order of seconds, minutes, and the length of task blocks (Chein et al., 2011; Tamnes et al., 2012; Medaglia et al., 2018), little is known about the extent to which processes related to the imbalance model vary within-person across intermediate time scales (e.g., days). Capturing these intermediate timescales will be important because different biopsychosocial antecedents may be implicated in the variability observed over distinct timescales. For example, in a study in which adults underwent 101 daily assessments of cognitive performance, trial-to-trial and day-to-day performance variability were unrelated to regional brain volume, while smaller prefrontal white matter volumes were associated with higher block-to-block variability (Lövdén et al., 2013). In contrast, variability at shorter timescales (e.g., day-to-day performance variability) may be related to fluctuations in stress and motivation (Sliwinski et al., 2006; Brose et al., 2010), highlighting the heterogeneous underpinnings of intraindividual variability across timescales. Notably, in the context of adolescent substance use, processes unfolding at daily timescales that may impact the functioning of cognitive control and socio-emotional systems (e.g., sleep disturbances, family, and peer relationships; Telzer et al., 2013a,b, 2014) may be more amenable to intervention (Fosco et al., 2013; Paavonen et al., 2016) than those processes unfolding over the course of years (e.g., development of white matter tracts; Asato et al., 2010). This fact underscores the importance of increased empirical research at these timescales.

Another important feature relating to the measurement of intraindividual variability is that it will allow important tests of within-person associations, moving from questions such as “Do adolescents who are high in sensation seeking engage in more drug use?” to “During moments or days when adolescents have greater sensation seeking tendencies than is typical for them, do they engage in more drug use than usual?” Critically, such an approach would allow us to align our methods to the within-person inferences that we often wish to make (Molenaar, 2004). It would also bring our measurement of imbalance model constructs in closer alignment with the timescale at which substance use – a discrete, episodic behavior – is occurring. Once an intensive time series of variables of interest (e.g., sensation seeking, cognitive control, number of cigarettes smoked, number of alcoholic beverages consumed) has been generated, within-person inferences can be made via the same disaggregation of between-person and within-person variation discussed in the context of intraindividual change (**Figure 2** middle).

Experience-sampling and daily diary designs, due to their capacity to capture individual functioning at daily and finer timescales, will be suited to examining processes at this intermediate timescale. Indeed, such designs have already provided insight into the importance of intraindividual variability in many constructs (primarily mood) for understanding adolescent substance use (Weinstein et al., 2008; Hedeker et al., 2009; Crooke et al., 2013). This line of research has provided insight into the antecedents and consequences of substance use and provides a template for how experience sampling and daily diary designs may be leveraged to test imbalance models of adolescent substance use (see Shiffman, 2009).

The feasibility of intensive longitudinal designs to capture intraindividual variability will rest on the availability of reliable measures for constructs of interest that can be deployed in daily life. Tasks that have been used extensively in the laboratory (i.e., the BART task) to assess adolescent risk-taking have recently been extended to assess risk-taking *in situ* (MacLean et al., 2018). Tasks assessing cognitive control have also been developed (Schuster et al., 2015) and have been used to assess fluctuations in performance in participants as young as 8 years of age (Dirk and Schmiedek, 2016). Measures to capture short-term variability in incentive processes are also available (Lindert et al., 2018). An important challenge to overcome is to establish that these measures can reliably capture within-person variability in adolescents (methods for this purpose are available; Cranford et al., 2006).

In addition to the challenges of developing measures sensitive to fluctuations in imbalance model constructs, attrition is a common concern due to the intensive measurement protocols that come with intensive longitudinal designs. However, compliance with *in situ* measurement is generally high in adolescents, even in adolescent samples for which low compliance may be expected (e.g., adolescent smokers undergoing smoking cessation; Gwaltney et al., 2008). Moreover, the emergence of passive data collection approaches (e.g., sensors integrated into watches, phones, bracelets, and other clothing; Nusser et al., 2006) will greatly reduce participant burden. Methods of passive data collection with promise for capturing constructs of interest include sensors measuring heart rate variability (Majumder et al., 2017), a notable index of cognitive control (Thayer et al., 2009).

## Matching Models and Methods: Describing the Individual

The extent of heterogeneity in the functioning of cognitive control and socio-emotional systems during adolescence is beginning to be appreciated (Foulkes and Blakemore, 2018). While increases in drug use occur during adolescence (Moss et al., 2014), *most* adolescents *do not* engage in drug use (Hooshmand et al., 2012). At the level of psychological traits, tremendous heterogeneity in the magnitude of change in sensation seeking and impulsivity across adolescence has been observed (Harden and Tucker-Drob, 2011). In regards to brain structure, Mills et al. (2014) observed the hypothesized earlier maturation of the nucleus accumbens relative to the prefrontal cortex in only 17 out of 33 participants with at least three scans in childhood through early adulthood, indicating substantial heterogeneity in the development of these structures. In terms of brain function, age typically accounts for a modest portion of the variance in ventral striatal response to rewards (Bjork et al., 2010) due to vast interindividual differences within age groups (Somerville et al., 2010).

In order to better capture the heterogeneity that is a particularly salient feature of adolescence, there is a great need for an influx of person-centered research (Molenaar, 2004; Molenaar and Campbell, 2009). Currently, data are generally aggregated across individuals but data from the group will only describe the functioning of any particular individual under very strict conditions (i.e., if participants come from a homogeneous sample). Intensive longitudinal methods, because they result in sufficiently dense time series to capture individual functioning, lend themselves to an idiographic approach that aims to make predictions about the individual through the analysis of within-person variation over time.

Recent analytic developments have leveraged the intensive longitudinal data available with fMRI. Each repetition time (TR) is treated as a data point within a time series often spanning 6 min or more in order to employ a person-specific approach that will allow us to have greater confidence that we are capturing individual adolescent functioning (Gates and Molenaar, 2012). For example, imbalance models highlight the importance of changes in connectivity among prefrontal and subcortical brain regions through adolescence for understanding changes in drug use. Yet, these hypothesized changes in connectivity are often not tested. Instead, general linear model analyses of group differences are employed, highlighting how the blood-oxygen-level-dependence activity in certain regions of the brain distinguishes adolescents from adults (e.g., Galvan et al., 2006; Chein et al., 2011). Novel connectivity methods such as extended unified structural equation modeling capitalize on the many data points collected during fMRI scanning. As a result, they are capable of estimating person-specific networks of functional connectivity among brain regions and have recently been showcased as providing a better alignment of imbalance models and the methods used to test them (see Beltz, 2018).

Notably, intensive longitudinal approaches capable of capturing heterogeneity in brain function and organization may also be applied to behavioral data. This is routinely done in adolescent research in general (e.g., Russell et al., 2016; Lydon-Staley et al., 2018) within the context of multilevel models that accommodate the nested nature (with measurement occasions nested within participants) of repeated measures data (Snijders and Bosker, 2012). In this approach, random effects may be specified for an independent variable, allowing its effect to vary across participants. This allows some degree of heterogeneity to be captured. We note, however, that heterogeneity from this perspective is modeled as deviations from the association observed in the prototypical individual. Further, the deviations are often assumed to follow a normal distribution, placing limits on the extent to which heterogeneity can be modeled. Alternative approaches, when sufficiently long time series (>20 or more; Beltz et al., 2016) are available, include the novel methods that are currently being used to model person-specific connectivity patterns among brain regions. When these methods are applied to behaviors rather than brain regions, the resulting networks indicate within-person associations among multiple behaviors across time (Wright et al., 2015; Beltz et al., 2016).

Once the challenges of collecting dense time series of constructs of interest to imbalance model researchers are overcome, we anticipate the emergence of studies in which timeseries of imbalance model constructs (e.g., cognitive control, sensation seeking) and processes likely to impact these functions (e.g., stress, family, and peer context) are collected through experience-sampling methods and modeled as dynamic, person-specific networks (**Figure 2** top). These studies will provide insight into how changes in cognitive control and sensation seeking augment or blunt substance use behaviors in a person-specific manner and how changes in cognitive control and sensation seeking are in turn influenced by changes in the broader psychosocial context.

## Conclusion

Imbalance models of adolescent risk-taking have the potential to provide insight into the processes underlying substance use and abuse. Use of intensive longitudinal designs, by collecting many more data points on individual participants than is typical of longitudinal panel designs, will allow an assessment of intraindividual change and intraindividual variability in individual adolescents. This will allow explicit testing of the within-person processes put forward by imbalance models of adolescent substance use and will also encourage greater consideration of the extent to which processes influencing adolescent substance use play out across multiple timescales. This will bring the field closer to understanding how intraindividual variability in the socio-emotional and cognitive control systems occurring at relatively fast timescales (e.g., changes in cognitive control accuracy in response to momentary changes in motivation; Geier et al., 2009) may be linked to both slow-changing (e.g., development of white matter tracts; Asato et al., 2010) and fast-changing processes (e.g., daily interparental conflict; Fosco and Lydon-Staley, 2017), and how this places adolescents at risk for engaging in substance use.

## Author Contributions

DML-S and DSB contributed to the writing of and approved the manuscript.

## Conflict of Interest Statement

The authors declare that the research was conducted in the absence of any commercial or financial relationships that could be construed as a potential conflict of interest.
